# Changes of regional brain activity following Tuina therapy for patients with painful cervical spondylosis: a resting-state fMRI study

**DOI:** 10.3389/fneur.2024.1399487

**Published:** 2024-09-13

**Authors:** Shilong Song, Yun Fang, Xing Wan, Lili Shen, Yidan Hu, Chao Lu, Tao Yue, Lu Chen, Jianhuai Chen, Mingxin Xue

**Affiliations:** ^1^Department of Tuina, Jiangsu Province Hospital of Chinese Medicine, Affiliated Hospital of Nanjing University of Chinese Medicine, Nanjing, China; ^2^Department of Radiology, Jiangsu Province Hospital of Chinese Medicine, Affiliated Hospital of Nanjing University of Chinese Medicine, Nanjing, China; ^3^Department of Acupuncture and Rehabilitation, Jiangsu Province Hospital of Chinese Medicine, Affiliated Hospital of Nanjing University of Chinese Medicine, Nanjing, China; ^4^Department of Andrology, Jiangsu Province Hospital of Chinese Medicine, Affiliated Hospital of Nanjing University of Chinese Medicine, Nanjing, China; ^5^First Clinical Medical College, Nanjing University of Chinese Medicine, Nanjing, China

**Keywords:** cervical spondylosis, pain, Tuina therapy, resting-state functional magnetic resonance imaging, regional homogeneity

## Abstract

**Background:**

The effectiveness of Tuina therapy has been confirmed in treating pain of patients with cervical spondylosis (CS), however, its therapeutic mechanism is still unclear. This study aimed to observe the changes of regional brain activity following Tuina therapy in patients with painful CS based on resting-state functional magnetic resonance imaging (rs-fMRI) data.

**Methods:**

A total of 27 patients with CS and 27 healthy subjects (HCs) were enrolled in this study. All patients received Tuina therapy every 2 days for 2 weeks. The clinical manifestations of patients were evaluated by the Visual Analog Scale (VAS) and Neck Disability Index (NDI) before and after treatment. In addition, rs-fMRI data were collected and preprocessed in all patients before and after treatment, as well as HCs. HCs underwent a 1-time rs-fMRI scan, whereas CS patients underwent 2-times of rs-fMRI scan. The measure of regional homogeneity (ReHo) was calculated and compared between groups. Finally, relationships between altered brain regions and clinical characteristics were evaluated by *Pearson’s* correlation analysis.

**Results:**

After Tuina therapy, VAS and NDI scores of patients decreased. Before treatment, CS patients showed higher ReHo values in the left middle temporal gyrus, left thalamus, right anterior and posterior cingulate gyrus, left inferior parietal gyrus and lower ReHo values in the right gyrus rectus when compared with HCs. After treatment, CS patients exhibited higher ReHo values in the left inferior temporal gyrus, right anterior and posterior cingulate gyrus, left inferior parietal gyrus and lower ReHo values in the right rectus gyrus when compared with HCs. CS patients after treatment demonstrated higher ReHo values in the left inferior occipital gyrus when compared with those before treatment. Positive correlations were found between ReHo values of the right rectus gyrus and VAS, NDI scores in CS patients before treatment. Differences of VAS scores between before and after treatment were negatively correlated with ReHo values of the left inferior temporal gyrus in CS patients after treatment.

**Conclusion:**

This study demonstrated the presence of asynchronous activity in certain brain regions in CS patients, which might be associated with pain and cervical spine dysfunction. Tuina therapy might modulate asynchronous activity of abnormal brain regions, which might contribute to the effectiveness of Tuina therapy in alleviating pain and cervical spine dysfunction in CS patients.

## 1 Introduction

Cervical spondylosis (CS) is a condition characterized by typical symptoms such as pain, numbness in the upper extremities and limited neck movement, and its primary clinical manifestation is pain (1). CS is now considered as a significant global public health burden, which is recognized as the fourth most disabling disease worldwide with an estimated annual global prevalence of over 30% (2). In 2020, a Global Burden of Disease survey reported a global age-standardized prevalence of 3551.1 cases and an incidence of 806.6 cases of neck pain per 100,000 people, respectively (3). It is essential to understand the sources of pain and the mechanism underlying the effectiveness of treatment. Biomechanical influences, such as osteophyte formation, disk degeneration, a decrease in the sagittal diameter of the spinal canal (4), inflammatory biomarkers including CRP, IL-1β, TNF-α, IL-6, etc. (5), as well as dysfunction in pain conduction and modulation at the level of the brain and spinal cord, are commonly associated with the pain symptom in patients with CS.

The central pathophysiological mechanism of pain in the cervical spine is complex. Previous study on the central mechanism of CS showed that the neurological symptoms were more pronounced in patients with CS (6). These symptoms were found to be negatively correlated with the activity in the precuneus, posterior cingulate gyrus and positively associated with the activity in the precentral gyrus, supplementary motor areas (6). Considering the multiple influencing factors, patients with CS might experience abnormal activation in multiple regions that caused the symptoms including pain, reduced neck movement and negative emotion. Resting-state functional magnetic resonance imaging (rs-fMRI) is a noninvasive technique to evaluate spontaneous brain activity of subjects during rest. Regional homogeneity (ReHo) is a commonly used parameter for measuring the level of local synchronization of intrinsic fMRI signals by calculating Kendall’s coefficient concordance (KCC) between the time series of a given voxel and those of its neighboring voxels (7). This method is commonly used to detect brain regions with altered activation in patients with neuropsychiatric disorders, as well as patients complained of pain (8–11). However, fewer studies have explored the central pathogenesis of CS using rs-fMRI. The utilization of neuroimaging methods to investigate the mechanisms of Tuina is currently being employed in multitude spinal disorders. For instance, Chen et al. examined the function of default mode network in the brain in patients with lumbar disk herniation and discovered that Tuina could modify the patient’s pain modulation patterns to achieve pain (12). Furthermore, rs-fMRI has been employed to investigate the analgesic effects of Tuina. Animal studies had demonstrated that the impact of Tuina on neuropathic pain relievers pain behavior by promoting cortical remodeling (13).

Various non-surgical protocols have been used to treat CS, including pharmacological treatments such as non-steroidal anti-inflammatory drugs (NSAIDs), selective 5-hydroxytryptamine reuptake inhibitors (SSRIs), opioids, sedative-hypnotic drugs, as well as non-pharmacological treatments such as physiotherapy, injections, surgical interventions (14, 15). However, the application of NSAIDs is limited by their adverse effects and poor tolerance (16). Considering the positive effects and safety, clinicians often recommend complementary therapies, including Tuina, acupuncture, exercise, yoga, tai ji, Chinese herbs and sports (17). The effectiveness of Tuina in the treatment of CS have been confirmed in numerous previous studies and the symptom of neck pain can be effectively relieved by Tuina therapy by raising the neck pain threshold (18–20). Proper release maneuvers can improve the tension of the cervical musculoskeletal nerves (21–23). Tuina therapy is now commonly used as a conservative treatment for CS. Manipulation practitioners employ a range of techniques to relax the cervical muscles and joints, including kneading, pressing, lifting and pulling. This is done in order to correct imbalanced joints and to relax tense and tired muscles. A randomized controlled trial study demonstrated that a combination of different manipulative techniques significantly improved pain symptoms and mobility function in patients with vertebral artery whiplash (24). A randomized controlled trial study of chronic neck pain by Kang et al. revealed that resistance exercise combined with massage achieved superior outcomes in terms of pain, cervical mobility, and trapezius muscle tone in patients with chronic neck pain (25). However, the therapeutic efficacy of Tuina is inconclusive due to its specificity, such as different therapeutic precision and methods, sample size issues, methodological quality and limitations of existing scientific tools in clinical studies of Tuina therapy.

In this study, we aimed to explore the differences in resting-state brain activity between CS patients and matched healthy controls (HCs), as well as the central mechanism underlying therapeutic effects of Tuina therapy in the treatment of CS. We hypothesized that (1) compared to HCs, CS patients might exhibit altered activity in specific brain regions that were linked to clinical symptoms of patients, and (2) after Tuina therapy, CS patients’ clinical symptoms might be improved, which might be achieved by the changes of spontaneous neural activity in these specific brain regions.

## 2 Participants and methods

### 2.1 Participants

The Ethics Committee of Jiangsu Province Hospital of Chinese Medicine, Affiliated Hospital of Nanjing University of Chinese Medicine approved this study (Ethics Approval No. 2022NL-169-02). In addition, it was registered in the China Clinical Trial Registration Center (registration number: ChiCTR2200066373), available.^1^ Moreover, all subjects gave their written informed consent to participate in the study. All patients were recruited from the Department of Tuina, Jiangsu Province Hospital of Chinese Medicine, Affiliated Hospital of Nanjing University of Chinese Medicine from September 2022 to December 2022. CS was diagnosed based on the 11th Revision of the International Classification of Diseases (ICD-11) (26) for CS and neck pain.

The inclusion criteria were as follows: (1) meeting the diagnostic criteria for CS; (2) aged 20–55; (3) right-handedness; (4) patients could either be treatment naive or had received prior therapy with a minimum washout period of 28 days; (5) those who were willing to be treated by Tuina therapy; (6) had good compliance and signed written informed consent.

The exclusion criteria were as follows: (1) neurogenic CS with surgical indications, excluding patients with spinal cord cervical spondylosis; (2) serious organic diseases and mental diseases; (3) patients with vertebral artery-type cervical spine vertigo episodes who were unable to take care of themselves; (4) patients with broken skin or other skin diseases at the site of Tuina treatment; (5) women who were breastfeeding, pregnant, or preparing to become pregnant; (6) patients with other chronic and persistent pain who needed to take pain medication; (7) patients participating in other clinical trials; (8) patients who had a fear of previous Tuina therapy or MRI examination or who suffered from claustrophobia.

### 2.2 Intervention of Tuina

The physician used a thumb of one hand and the four remaining fingers of the other hand, to apply pressure at Tuina acupoints on both sides of the neck muscles, including Jingbailao (EX-HN14), Fengchi (GB20) and Wangu (GB12). Additionally, longitudinal vertical flicking techniques were used on the neck muscles in the direction of their fibers for 5–6 repetitions. The physician applied Tuina manipulation on the neck and shoulder muscles, and repeated Tuina treatment 5–6 times, and used kneading techniques on various muscle groups in the scapular region of the patient’s back. Furthermore, acupressure points such as Bingfeng (SI12), Quqiguan (SI13), Shoulder Well (GB21) and Tianzong (SI11) were pressed and held, and the patient’s comfort level was considered to determine the optimal length of the treatment. The treatment lasted about 15–20 min and was done 2–3 times per week, with a one-day break in between, for a total of 6 treatments over 2 weeks.

### 2.3 Assessment measures

The Visual Analog Scale (VAS) was used to evaluate the level of pain in the cervical spine area, with a score of 0 representing the lowest level of pain and a score of 10 representing the highest level of pain. The Neck Disability Index (NDI) was used to evaluate the degree of cervical spine dysfunction based on 10 aspects, including pain intensity, reading, heavy lifting, headache, work, sleep, driving, recreational activities, concentration and self-care aspects of life.

### 2.4 MRI data acquisition and preprocessing

MRI data were acquired using a 3.0 T GE MRI scanner. All participants were instructed to stay relaxed and awake, keep eyes closed, not think of anything particular during the entire MRI scanning procedure. T1-weighted structural data were acquired using the following parameters: repetition time (TR) = 7.7 ms; echo time (TE) = 3.1 ms; slice thickness = 1 mm; field of view (FOV) = 256 × 256 mm^2^; matrix = 256 × 256; number of slices = 160. All rs-MRI data were acquired using the following parameters: TR = 2000 ms; TE = 30 ms; slice thickness = 3.5 mm; FOV = 224 × 224 mm^2^; matrix = 80 × 80; number of slices = 33; number of volumes = 240. Based on MATLAB, MRI data preprocessing was performed using the software of Data Processing Assistant for Resting-State fMRI (DPARSF) (27) according to the steps presented in our previous study. Subjects with head-translation more than 2.0 mm or rotation more than 2.0° were excluded in this study. In order to prevent other factors from interfering with the collection of rs-fMRI data, all participants were instructed on their body and emotional state, and were asked to maintain a still head and body, close their eyes, and refrain from thinking about anything. They were also instructed to remain relaxed.

### 2.5 ReHo calculation

The steps of ReHo calculation were as follows: (1) linear detrending; (2) temporal band-pass filtering; (3) regress out nuisance covariates. Finally, Kendall’s coefficients of concordance (KCC) were calculated by the correlations between the time series of a voxel and those of its 26 nearest neighbor voxels in a voxel-wise manner, which were defined as ReHo values. For standardization purpose, ReHo values were transformed to zReHo values by *Fisher’s r-to-z* transformation. Finally, all zReHo values were smoothed. ReHo reflects the synchronization of spontaneous brain activity within a region by measuring the consistency of a given voxel and its adjacent voxels. ReHo can evaluate the activity levels of brain regions and reflect the consistency of local neuronal activity levels. An increase in ReHo represents an increase in the consistency of local brain neuronal activity, while a decrease in ReHo indicates a decrease.

### 2.6 Statistical analysis

In this study, between-group differences in demographic and clinical data were compared using independent two-samples *t*-tests (normally distributed data), non-parametric tests (non-normally distributed data) and chi-squared tests (count data) by SPSS 25.0 software. *p* < 0.05 was considered statistically significant. In addition, between-group differences in ReHo values were compared using *t*-tests by the software of Resting-State fMRI Data Analysis Toolkit (REST) (28). The methods of GRF (voxel-level *p* < 0.001 and cluster-level *p* < 0.05) and AlphaSim (*p* < 0.001 and minimum cluster size was set at six voxels) methods in REST software were used for the correction of multiple comparisons. Finally, relationships between altered brain regions and clinical characteristics were evaluated by *Pearson’s* correlation analysis. *p* < 0.05 was considered statistically significant.

## 3 Results

### 3.1 Comparison of demographic and clinical data

No significant differences were observed in the gender, age and BMI between CS patients and HCs (Table 1). After treatment, CS patients exhibited decreased VAS and NDI scores when compared with those before treatment (Table 2).

**Table 1:** Demographic and clinical characteristics between groups.

	CS (*n* = 27)	HCs (*n* = 27)	*χ*^2^/*t*	*p*
Gender (M/F)	7/20	6/21	0.10	0.75
Age (years)	37.29 ± 14.60	39.00 ± 14.26	−0.43	0.67
BMI (kg/m^2^)	21.68 ± 3.20	21.14 ± 2.72	0.66	0.51

CS, cervical spondylosis; HCs, healthy controls. BMI, body mass index.

**Table 2:** Comparison of VAS and NDI scores before and after treatment CS.

	Before treatment (*n* = 27)	After treatment (*n* = 27)	*t*	*p*
VAS scores	4.74 ± 1.89	1.85 ± 1.91	8.126	<0.001
NDI scores	13.77 ± 6.15	5.29 ± 4.22	7.564	<0.001

CS, cervical spondylosis; VAS, visual analog scale; NDI, neck disability index.

### 3.2 Comparison of ReHo values between groups

**Figure 1:**
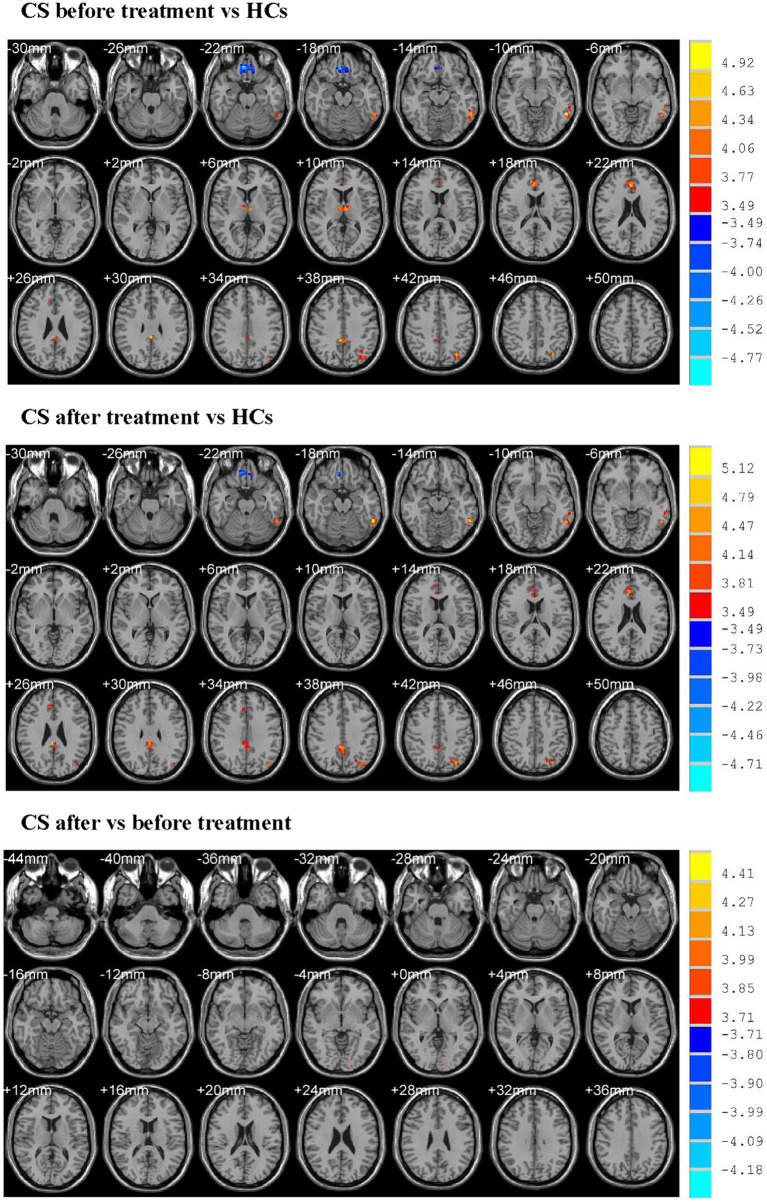
Showed altered ReHo values. CS, cervical spondylosis; HCs, healthy controls. ReHo, regional homogeneity.

Before treatment, CS patients showed higher ReHo values in the left middle temporal gyrus, left thalamus, right anterior and posterior cingulate gyrus, left inferior parietal gyrus and lower ReHo values in the right gyrus rectus when compared with HCs (Table 3; Figure 1).

**Table 3:** Comparison of ReHo values between groups.

Brain regions	Peak MNI coordinate			Cluster	Peak T value
	x	y	z		
CS before treatment vs. HCs^a^					
Left middle temporal gyrus	−57	−48	−9	73	5.20
Left thalamus	−3	−9	9	37	4.80
Right anterior cingulate gyrus	3	36	18	57	5.09
Right posterior cingulate gyrus	3	−36	27	39	4.74
Left inferior parietal gyrus	−33	−72	42	35	4.75
Right gyrus rectus	6	42	−21	90	−5.03
CS after treatment vs. HCs^a^					
Left inferior temporal gyrus	−60	−51	−15	75	5.45
Right anterior cingulate gyrus	6	36	24	44	4.76
Right posterior cingulate gyrus	3	−36	27	72	5.21
Left inferior parietal gyrus	−33	−72	42	44	4.87
Right rectus gyrus	9	42	−21	42	−4.70
CS after vs. before treatment^b^					
Left inferior occipital gyrus	−18	−96	−6	6	4.55

CS, cervical spondylosis; HCs, healthy controls. ReHo, regional homogeneity; MNI, Montreal Neurological Institute; x, y and z, the coordinates of peak voxels of clusters in MNI space.

a GRF (voxel-level *p* < 0.001 and cluster-level *p* < 0.05) in REST software was utilized for the correction of multiple comparisons.

b AlphaSim (*p* < 0.001 and minimum cluster size was set at 6 voxels) in REST software was utilized for the correction of multiple comparisons.

After treatment, CS patients exhibited higher ReHo values in the left inferior temporal gyrus, right anterior and posterior cingulate gyrus, left inferior parietal gyrus and lower ReHo values in the right rectus gyrus when compared with HCs (Table 3; Figure 1).

CS patients after treatment demonstrated higher ReHo values in the left inferior occipital gyrus when compared with those before treatment (Table 3; Figure 1).

### 3.3 Relationships between altered brain regions and clinical characteristics

**Figure 2:**
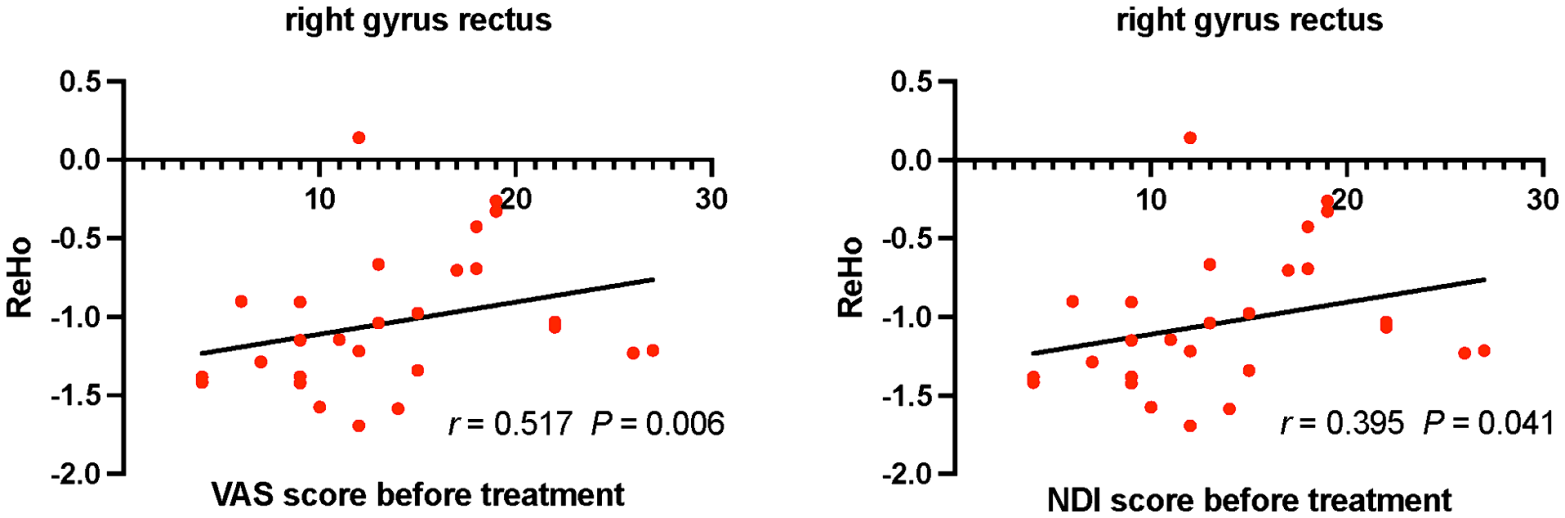
Relationships between VAS, NDI scores and ReHo values in CS patients before treatment. CS, cervical spondylosis. ReHo, regional homogeneity. VAS, visual analog scale; NDI, neck disability index.

**Figure 3:**
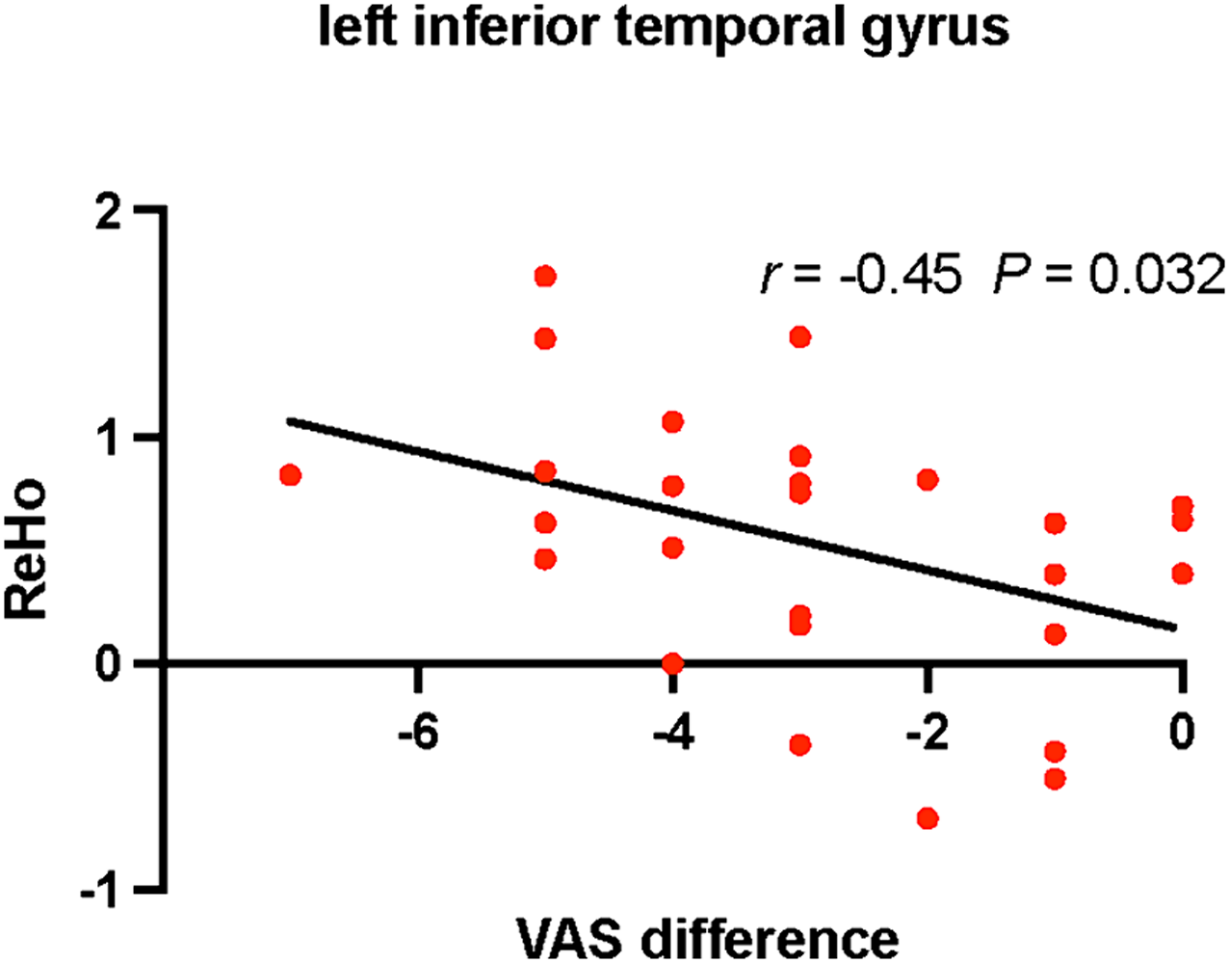
Relationships between differences of VAS scores and ReHo values in CS patients after treatment. CS, cervical spondylosis. ReHo, regional homogeneity. VAS, visual analog scale.

Positive correlations were found between ReHo values of the right rectus gyrus and VAS (*r* = 0.517, *p* = 0.006), NDI (*r* = 0.395, *p* = 0.041) scores in CS patients before treatment (Figure 2). Differences of VAS scores between before and after treatment were negatively correlated with ReHo values of the left inferior temporal gyrus in CS patients after treatment (*r* = −0.45, *p* = 0.032; Figure 3).

## 4 Discussion

To the best of our knowledge, this was the first rs-fMRI study exploring the central pathological mechanism of CS and the changes of brain activation associated with Tuina therapy in the treatment of CS. Compared with HCs, CS patients before treatment showed increased consistency of spontaneous neural activity in the left middle temporal gyrus, left thalamus, right anterior and posterior cingulate gyrus, left inferior parietal gyrus, as well as decreased consistency of neural activity in the right gyrus rectus. After treatment, CS patients exhibited higher activity in the left inferior temporal gyrus, right anterior and posterior cingulate gyrus, left inferior parietal gyrus and lower activity in the right rectus gyrus when compared with HCs. Moreover, CS patients after treatment demonstrated higher activity in the left inferior occipital gyrus when compared with those before treatment. Positive correlations were found between ReHo values of the right rectus gyrus and VAS, NDI scores in CS patients before treatment. Differences of VAS scores between before and after treatment were negatively correlated with ReHo values of the left inferior temporal gyrus in CS patients after treatment.

Previous MRI study showed that neck pain could cause reduced gray matter volume in the right middle cingulate cortex, right superior temporal gyrus, right precuneus in patients with chronic cervical spondylotic pain and these patients displayed decreased functional connectivity between the right precuneus and bilateral medial prefrontal cortex (29). Additionally, gray matter volume of the right middle cingulate cortex, right superior temporal gyrus and right precuneus, as well as resting-state functional connectivity between the right precuneus and bilateral medial prefrontal cortex, were negatively correlated with the VAS respectively (29). Another rs-fMRI study demonstrated that CS patients had altered amplitude of low-frequency fluctuation (ALFF) in the middle cingulate cortex, cerebellum and middle frontal gyrus (30). Previous studies demonstrated that pain could cause extensive functional alterations in several brain networks, particularly in the default mode network (DMN) (31, 32). The DMN is composed of several brain regions, including the medial prefrontal cortex, inferior parietal lobule, posterior cingulate gyrus/precuneus, hippocampus, angular gyrus and temporal lobe (33, 34). These regions exhibit strong neural activity during resting-state and decreased synchronized intrinsic neuronal activation during goal-oriented tasks (35, 36). Recurrent patients with chronic pain showed abnormal functional connectivity in DMN, which suggested that patients’ recurrent pain states were associated with enhanced spontaneous neural activity in the thalamus, temporal cortex, cingulate cortex (32).

Abnormal brain areas attributed to DMN, such as the thalamus, posterior cingulate gyrus, middle temporal gyrus and inferior temporal gyrus, were also identified in this study. The middle temporal gyrus is considered as a key region of DMN (37), and it is also a region involved in language and word processing, emotion and memory processing (38, 39). The middle temporal gyrus can integrate cognitional and emotional information under nociceptive stimulation and can express the degree of pain perception under the control of cortical–limbic system (40). The inferior temporal gyrus is an important region for cognition and learning (41). The left inferior parietal gyrus is a component of the frontoparietal network, and it is more specialized in the semantic encoding of retrieval of scene memories and executive processing of perceptual motions compared to the right inferior parietal gyrus (42). The anterior cingulate gyrus, which is a brain region associated with sensory and pain modulation, plays a role in receiving and storing painful emotional information. Pain triggers the activation of anterior cingulate gyrus to improve the transmission of sensory-motor neurons (42–46). Suppressing the activity in the anterior cingulate gyrus could lead to a reduction in pain and adverse emotion (45). The anterior and posterior cingulate gyrus are critical constituents of the limbic system (47). The posterior cingulate gyrus is a brain region that possesses several features, including sensory monitoring, spatial localization, internal orientation and high metabolic rate (48). Additionally, it receives nociceptive information from the thalamus, which contributes to its sensitivity. In addition, the rectus gyrus, located on the orbital surface of the frontal lobe, is a part of the limbic system and medial prefrontal brain network. This region is the main origin of downstream connections from the medial prefrontal lobe to the hypothalamus, brainstem, and it is a part of the limbic system and DMN, which plays a key role in transmitting information in the brain (47, 49).

The thalamus, anterior cingulate gyrus, posterior cingulate gyrus, insula, amygdala and periaqueductal gray matter are considered as “pain matrix,” indicating that injurious nociceptive stimuli can exert feedback modulation on these regions (50–52). Following 2 weeks of Tuina therapy, CS patients exhibited higher ReHo values in the left inferior temporal gyrus, right anterior and posterior cingulate gyrus, left inferior parietal gyrus and lower ReHo values in the right rectus gyrus when compared with HCs. Increased ReHo values of the anterior cingulate gyrus after treatment suggested increased compensatory inhibition of the nociceptive neural circuit associated with the thalamic-cingulate cortex, which might lead to a reduction in pain sensation. Furthermore, compensatory spontaneous neural activity was observed in the posterior cingulate gyrus due to the reduction in pain sensation. However, the thalamus disappeared in the differences of brain regions between CS patients and HCs. The thalamus is the relay station for information rectification and transmission of injurious nociceptive stimulus in the brain, which can feedback activate the thalamus (53, 54). These findings suggested that inhibition of the nociceptive neural circuit involving the thalamus-cingulate cortex, resulting in a reduction in pain sensation. Therefore, we speculated that the thalamus was an important relay station for pain information transmission, and the cingulate cortex, temporal lobe cortex were the key regions for encoding information about injurious nociceptive transmitted upstream from the thalamus in CS patients with neck pain.

The study confirmed the ameliorative effect of Tuina therapy on pain and cervical spine dysfunction in patients with CS. The changes in the ReHo values of the right rectus gyrus and left inferior temporal gyrus, in conjunction with the improvement in symptom scores observed prior to and following Tuina therapy, provided evidences that the analgesic effect and relief of cervical spine dysfunction could be fed back into the central nervous system through brain area activity. However, the only brain area that displayed differences in ReHo values before and after treatment was the left inferior occipital gyrus. The small number of differences observed in brain areas might be due to the selection of right-handed subjects, along with a small sample size and the shorter treatment period, which were the two limitations of this study. The results of this study were partly inconsistent with previous studies, but there were some similarities and overlaps. Possible explanations for this could be that CS itself was a clinical symptom manifestation and it had several subtypes. Additionally, previous studies had smaller and mostly observational samples, which might have contributed to the differences in results. Therefore, future prospective longitudinal follow-up and multimodal neuroimaging studies with larger sample sizes were needed to further validate these findings, as well as other objective indicators including inflammatory biomarkers and additional imaging techniques like PET or EEG in patients with different subtypes of CS. Moreover, a negative correlation was found between changes in VAS scores and ReHo values in the left inferior temporal gyrus. However, the single finding of a negative correlation between VAS score changes and ReHo values was not sufficient to establish a definitive relationship. The joint evaluation of these multiple indicators (brain structure, brain electrophysiological and metabolic activity, inflammatory biomarkers) might better establish a definitive relationship.

## 5 Conclusion

In conclusion, CS patients showed impaired activity in the pain-related brain regions, resulting in neck pain and limited neck movement. Tuina therapy could improve the clinical conditions of pain and cervical mobility, which might be achieved by modulating asynchronous activity of abnormal brain regions. All these findings provided new insights into understanding the central mechanism underlying the therapeutic effects of Tuina in the treatment of CS.

## Data Availability

The raw data supporting the conclusions of this article will be made available by the authors, without undue reservation.

## Footnotes

^1^https://www.chictr.org.cn/
